# Double trouble in device infection: Concomitant transvenous and leadless pacemaker infection

**DOI:** 10.1016/j.hrcr.2026.06.029

**Published:** 2026-06-30

**Authors:** Justin Phan, Nasser Al Solaia, Alper Aydin, Jamal Alkadri, Mehrdad Golian

**Affiliations:** 1 Department of Cardiac Electrophysiology, University of Ottawa Heart Institute, Ottawa, Ontario, Canada; 2 Department of Anesthesia, University of Ottawa Heart Institute, Ottawa, Ontario, Canada

**Keywords:** Cardiac implantable electronic device infection, Leadless pacemaker, Transvenous pacemaker, Endocarditis, Lead extraction

**Key Teaching Points**

• Despite a lower risk of infection, leadless pacemakers may also become infected, and a high index of suspicion should be maintained in patients presenting with bacteremia.
• Leadless pacemaker endocarditis may involve the tricuspid subvalvular apparatus, which may have implications for antibiotic duration and timing of re-implant procedures.
• Transesophageal and intracardiac echocardiography may be useful adjuncts in the removal of infected leadless pacemakers.

## Introduction

Leadless pacemakers (LPMs) are an increasingly used form of cardiac implantable electronic device (CIED) in patients requiring permanent pacing, as they offer a minimally invasive option and eliminate lead-related and generator-related complications associated with transvenous pacemakers. 1 significant advantage of LPMs is the lower risk of device infection compared to transvenous devices. Garweg et al^1^ found no cases of infection in patients implanted with the Micra (Medtronic, Minneapolis, MN) LPM, even in patients with active bacteremia. Ip et al^2^ found a lower rate of infection with the Aveir VR (Abbott, Chicago, IL) compared to single-chamber transvenous pacemakers in a Medicare database (0.4% vs 1.4%, *P* = .002). Nevertheless, LPM infections have been described in patients who present with bacteremia, such as in the recent case report of an infected Aveir atrial LPM in a patient with methicillin-resistant *Staphylococcus aureus* bacteremia.^3^ We describe an interesting case of device infection in a patient who underwent the insertion of an Aveir LPM to mitigate the risk of radiotherapy to a pre-existing left-sided transvenous pacemaker.

## Case report

A 68-year-old man was transferred to our institution in December 2025 after presenting with fever of unknown origin. Blood cultures were performed, with 4 culture bottles returning positive for methicillin-sensitive *S. aureus* (MSSA).

He had a complex device history with insertion of a dual chamber transvenous pacemaker in 2016 for complete atrioventricular block. In April 2025, he had been diagnosed with a left neck adenocarcinoma of unknown primary. As the patient required radiotherapy to his left neck and there radiotherapy-induced CIED dysfunction was not a concern, he underwent the insertion of an Aveir VR LPM in June 2025. The LPM was programmed VVI 40 to allow for backup pacing in the event of malfunction of his transvenous pacemaker. He subsequently underwent radiotherapy to the left neck and axilla (40 Gray) without complication. He had ongoing treatment with combination chemotherapy (carboplatin/paclitaxel).

**Figure 1:**
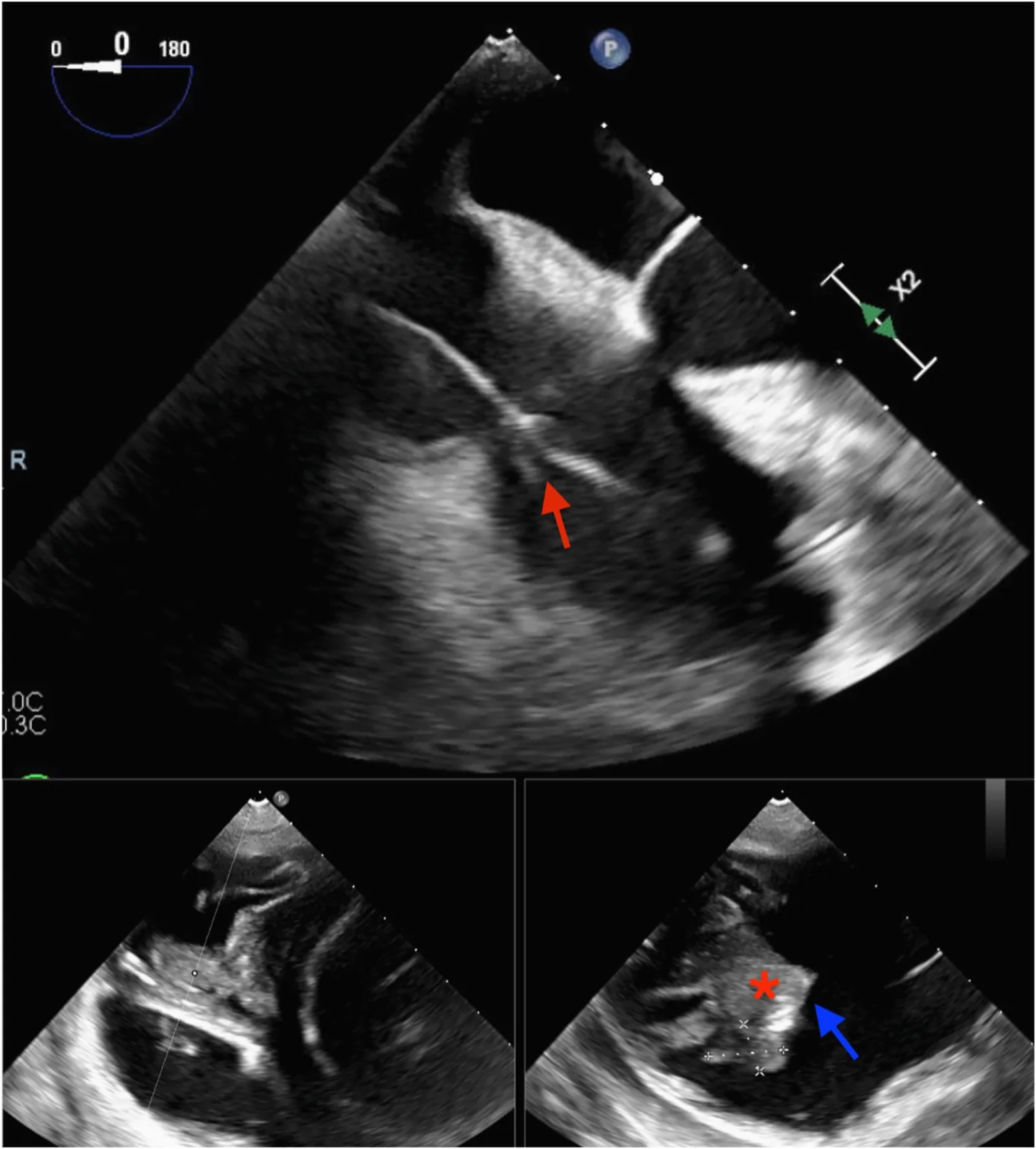
Transesophageal echocardiogram showing infective vegetations on both cardiac implantable electronic devices. (*Red arrow*) Infective vegetation on the right atrial portion of the ventricular lead. (*Red star*) Body of the leadless pacemakers. (*Blue arrow and markers*) Associated infective vegetation on the leadless pacemakers and subvalvular apparatus.

In the current presentation, cefazolin 2 g TID was started, and transthoracic echocardiography was performed, which did not show obvious evidence of CIED infection. A follow-up transesophageal echocardiogram demonstrated vegetations on the right atrial portion of the ventricular lead and the LPM, confirming device infection affecting both devices (Figure 1). The patient was subsequently transferred to our institution for device extraction.

**Figure 2:**
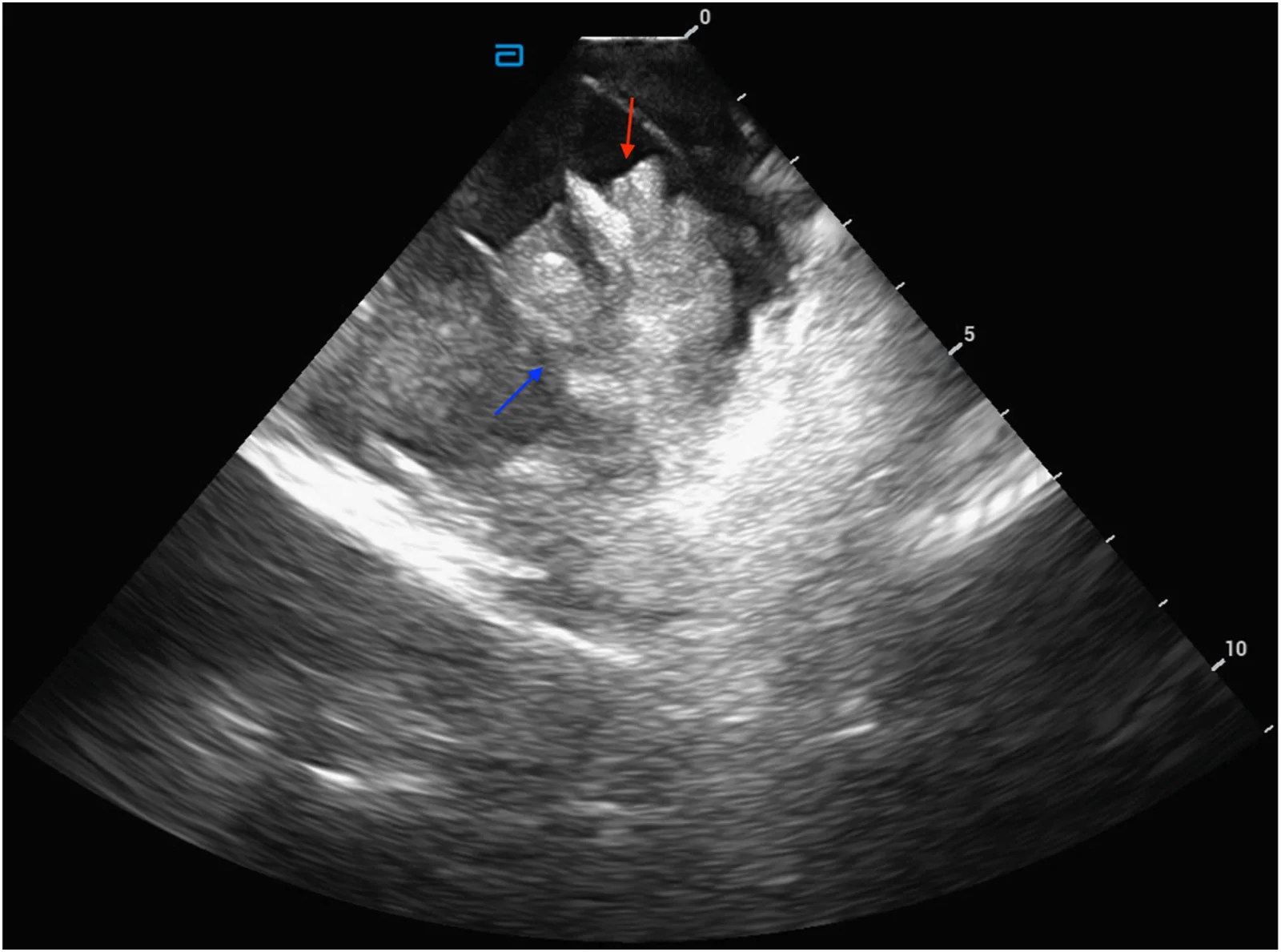
Intracardiac echocardiogram of the right ventricle showing the leadless pacemaker (*blue arrow*) and associated infective vegetation (*red arrow*), with probable involvement of the surrounding papillary muscle.

The patient was taken for emergent device extraction on the day of transfer. The procedure was performed under general anesthesia. Bilateral femoral venous access was obtained, through which a temporary pacemaker was introduced to the right ventricular apex and a 9-French intracardiac echocardiography (ICE) catheter (ViewFlex, Abbott, Chicago, IL) was inserted into the right atrium. ICE confirmed a vegetation on the LPM with probable involvement of the underlying papillary muscle (Figure 2). Following this, the left-sided transvenous device pocket was opened, and the leads were dissected out. EZ lead-locking devices (Philips, Amsterdam, The Netherlands) were inserted into each lead. Using a 16-French laser extraction sheath (Spectranetics, Fremont, CA), the right ventricular lead was removed, followed by the right atrial lead. ICE confirmed no pericardial effusion. The left-sided pocket was irrigated and closed.

**Figure 3:**
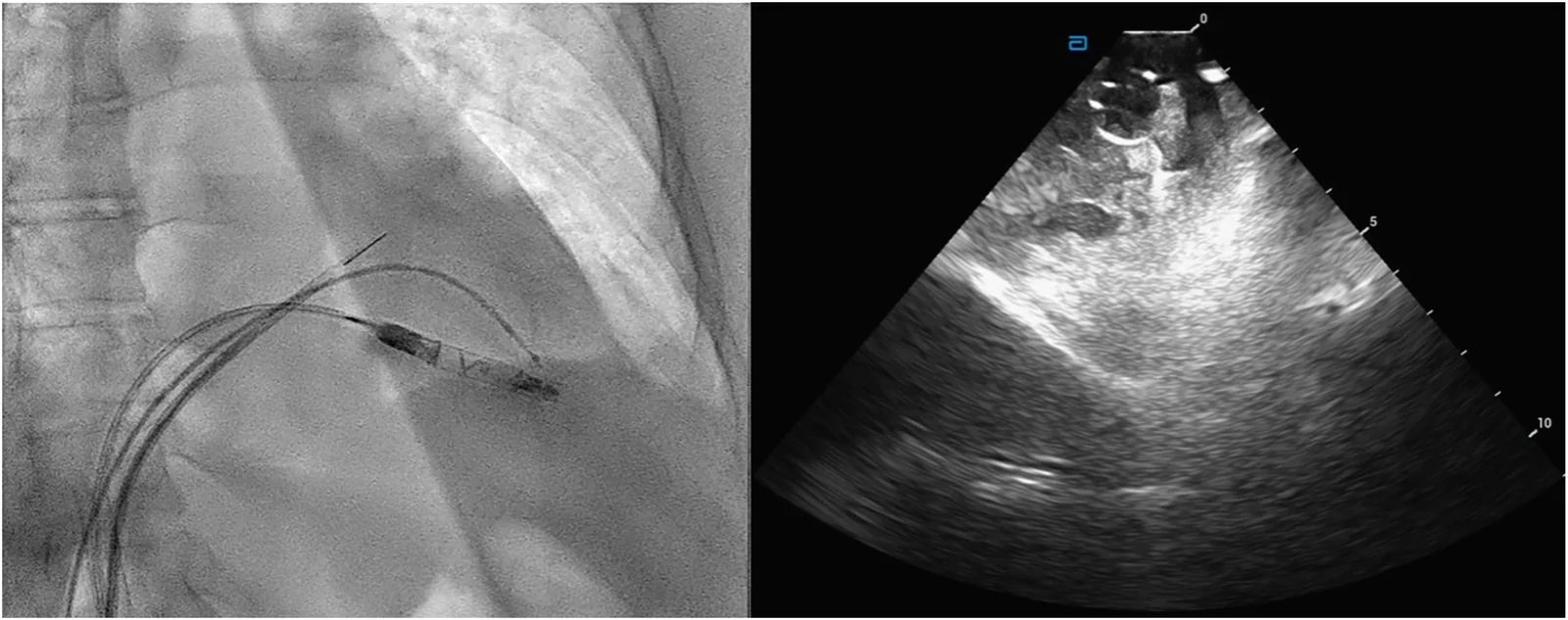
Fluoroscopic and intracardiac echocardiogram showing snaring of the leadless pacemaker.

Following this, the right femoral access site was upsized to a 26-French Aveir Retrieval Sheath (Abbott, Chicago, IL). Using a combination of ICE and fluoroscopic guidance (Figure 3), the hub of the Aveir LPM was snared and docked into the retrieval sheath. Using counterclockwise rotation, the device was freed from its fixation site, retracted into the sheath, and removed from the body. Examination of the LPM *ex vivo* demonstrated adherent infective material/vegetation and was sent for culture. ICE confirmed no pericardial effusion, although there appeared to be residual infected vegetation on the right ventricular papillary muscle/subvalvular tricuspid apparatus.

Ultrasound guidance was then used to obtain right axillary access. A 7-French splitable sheath was then inserted, and an active fixation lead (Medtronic 5076, Minneapolis, MN) was introduced to the right ventricle and fixated under fluoroscopic guidance. Pacing parameters were satisfactory, and the lead was secured to the skin. A single-chamber pacemaker (Medtronic Astra XT SR) was attached. The lead and generator were secured to the skin for use as a “temporary-permanent pacemaker.” The femoral venous access sites were then closed using suture-mediated closure devices (Perclose, Abbott, Chicago, IL), and the procedure was concluded.

**Figure 4:**
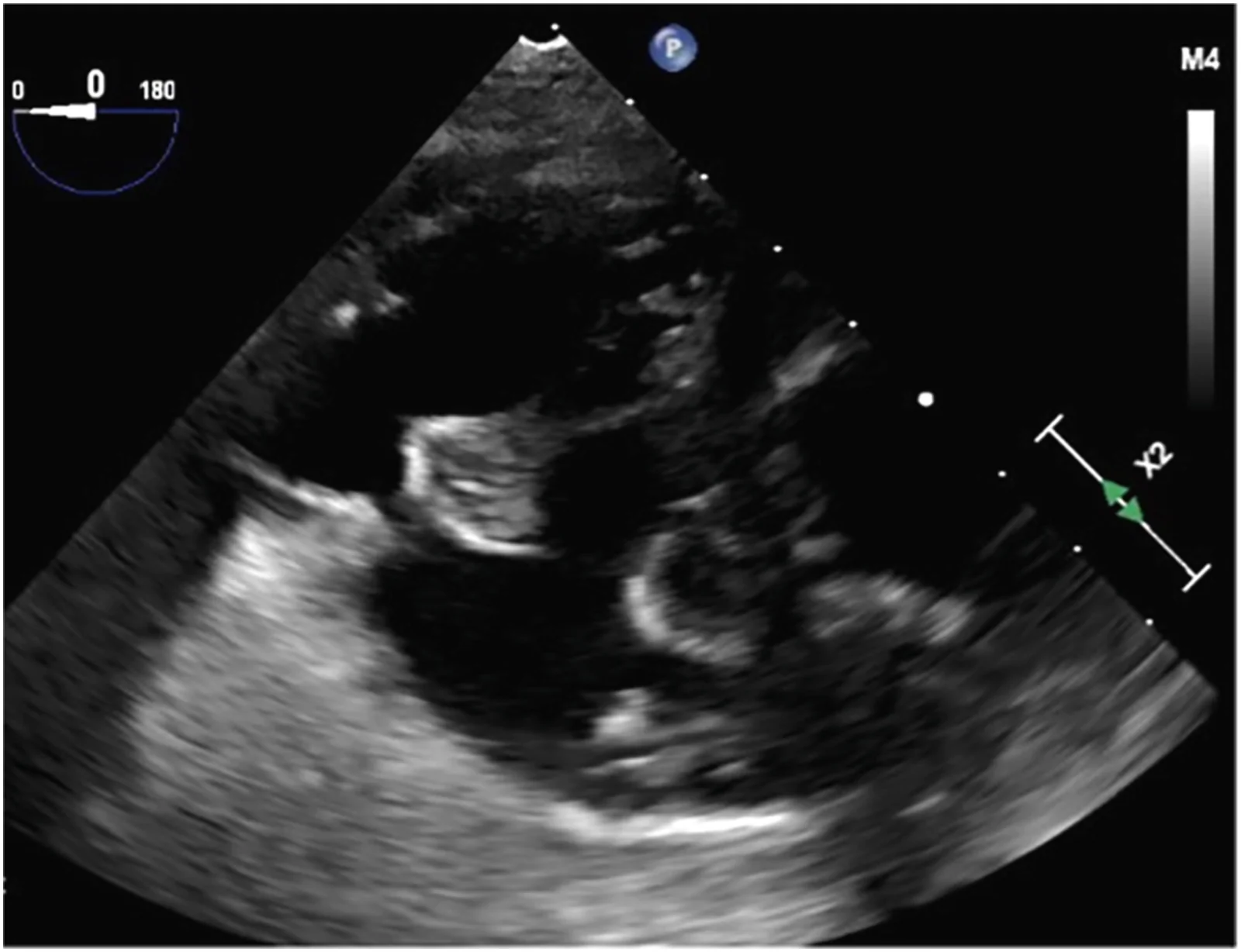
Follow-up transesophageal echocardiogram showing resolution of previous infected vegetations on the tricuspid subvalvular apparatus.

Following his device extraction, all subsequent blood cultures were negative. There was no growth from the infective material on the LPM. Intravenous cefazolin was continued for 6 weeks. Follow-up transesophageal echocardiography demonstrated resolution of the residual right ventricular vegetations (Figure 4). The patient opted to be discharged with a temporary–permanent pacemaker rather than undergo reimplant due to a limited prognosis from his underlying cancer.

## Discussion

Our case demonstrates a rare case of a patient with concomitant infection of a transvenous pacemaker and LPM. To the best of our knowledge, only 1 other case of a patient with LPM infection and concomitant transvenous leads has been reported by Okada et al.^4^ In contrast to the case presented by Okada et al,^4^ our patient did not have his LPM implanted in the setting of an infected transvenous device where only the generator was explanted, but the leads were left *in s*itu. In our case, the patient developed a *de novo* infection of both devices, with his only major risk factors being chemotherapy and cachexia. He did not have other risk factors for CIED infection, such as diabetes, chronic kidney disease, or other recurrent infections.

A further interesting aspect of our case is the involvement of the subvalvular apparatus, which was seen on ICE imaging. This resolved with prolonged antibiotic therapy, which would be considered a prerequisite should this patient undergo device reimplant. A previous case series of LPM infections^5^ found 8 cases, in which LPM vegetations were noted in 7 of 8 cases, although the involvement of the subvalvular apparatus remains unclear.

Percutaneous mechanical debulking (PMD) of vegetations using a mechanical aspiration device (AlphaVac, AngioDynamics, NY) has been described as an adjunctive technique in patients undergoing CIED extraction with large vegetations.^6^ Malyshev et al^6^ showed, in a case series of 42 patients (13 with PMD), that patients who had adjunctive PMD at the time of extraction had a lower mortality compared to those without (0 vs 4 patients). This approach may have been useful in debulking the subvalvular vegetation; however, the PMD tool is not widely available, and its use in LPM infections has not been reported to our knowledge.

LPMs, compared to transvenous PMs, demonstrate an apparently lower infection risk. In the 1,801 implants of the Micra leadless pacemaker (Medtronic, MN) from the Investigational Device Exemption (IDE) and Post-Approval Registry (PAR), there was only 1 infected device (0.002%), which was successfully managed by device extraction.^7^ The same group has shown a low rate of LPM infection (0/105 patients) even when LPMs were reimplanted in patients with CIED infections.^8^ There are a number of potential mechanisms for the lower risk of infections with LPMs compared to transvenous CIEDs. LPMs do not require the insertion of a device in a subcutaneous pocket with long leads traversing the vasculature. Pocket infection is the most common presentation of CIED infection (approximately 60%)^9^ with the most common mechanism being due to bacterial contamination during implant.^10^ Furthermore, the surface area of LPMs (∼616 mm^2^ for Micra) is much smaller than transvenous devices (∼3500 mm^2^ for a standard pacing lead), which may result in a lower risk of bacterial seeding.^11^ The material construction of LPMs may also contribute to a lower risk of infection. The Micra LPM is made from titanium coated with a layer of parylene, which has been shown to reduce the ability of bacteria to grow in colony count assays compared to bare titanium surfaces.^12^ Nevertheless, our case shows that LPM infections may still occur and should be considered in all patients who present with bacteremia and a high-risk organism, such as *S. aureus*.

## Conclusion

Leadless pacemakers are increasingly used for permanent pacing. Although device infection is rare, early diagnosis and device extraction are critical in ensuring optimal outcomes. Involvement of the subvalvular apparatus may occur, which may require prolonged antibiotic treatment and repeat imaging to ensure resolution before reimplant.

## Funding Sources

This research did not receive any specific grant from funding agencies in the public, commercial, or not-for-profit sectors.

## Disclosures

The authors have no conflicts of interest to disclose.
